# Vector Competence of Thrips Species to Transmit Soybean Vein Necrosis Virus

**DOI:** 10.3389/fmicb.2019.00431

**Published:** 2019-03-19

**Authors:** Jinlong Han, Vamsi J. Nalam, I-Chen Yu, Punya Nachappa

**Affiliations:** ^1^Department of Entomology and Plant Pathology, North Carolina State University, Raleigh, NC, United States; ^2^Department of Bioagricultural Sciences and Pest Management, Colorado State University, Fort Collins, CO, United States; ^3^Department of Anatomy and Cell Biology, Indiana University School of Medicine, Fort Wayne, IN, United States

**Keywords:** soybean vein necrosis virus, thrips, vector competence, immunolabeling, confocal microscopy

## Abstract

Soybean vein necrosis virus (SVNV) is a newly discovered species of tospovirus infecting soybean plants that is transmitted by the primary vector, soybean thrips (*Neohydatothrips variabilis*), and two additional secondary vectors, tobacco thrips (*Frankliniella fusca*) and eastern flower thrips (*F. tritici*). This study was undertaken to elucidate the association between virus acquisition [6, 12, 24, and 48 h acquisition access period (AAP)] and transmission efficiency [12, 24, and 48 h inoculation access period (IAP)] in the primary vector, *N. variabilis*, and to examine the mechanisms of vector competence by analyzing the effect of AAP (6, 12, and 24 h) on virus infection in various tissues. In addition, we examined virus infection in tissues of the two secondary vectors. We found a significant effect of virus acquisition on transmission efficiency, transmission rate post 6 and 48 h AAP was significantly lower than 12 and 24 h AAP. Our analysis did not reveal a correlation between virus transmission rate and virus RNA in corresponding *N. variabilis* adults. On the contrary, *N. variabilis* adults harboring higher accumulation of the virus (>10^4^) resulted in lower transmission rates. Analysis of SVNV infection in the tissues revealed the presence of the virus in the foregut, midgut (region 1, 2, and 3), tubular salivary glands and principal salivary glands (PSG) of adults of all three vector species, however, the frequency of infected tissues was highest in *N. variabilis* followed by *F. fusca* and *F. tritici*. The frequency of SVNV infection in individual tissues specifically the salivary glands was lowest after 6 h AAP compared to 12 and 24 h AAP. This finding is in agreement with the transmission assays, where significantly lower virus transmission rate was observed post 6 h AAP. In addition, *N. variabilis* adults with high PSG infection (12 and 24 h AAP) were likely to have high percentage of foregut and midgut region 2 infection. Overall, results from the transmission assays and immunolabeling experiments suggest that shorter AAP results in reduced virus infection in the various tissues especially PSG, which are important determinants of vector competence in SVNV-thrips interaction.

## Introduction

Soybean [*Glycine max* (L.) Merr.] is an important crop throughout the world due to its versatility as a food source along with applications in various industries such as textiles and biofuels. The United States is the global leader in soybean production with approximately 83 million acres planted and an estimated 117 million metric tons produced in 2017 (Soystats, 2017). There are several viral diseases that affect soybean crops in the United States. For instance, alfalfa mosaic virus, soybean mosaic virus and bean pod mottle virus cause significant yield reductions, decreased pod and seed set, reductions in oil content of seeds, and seed discoloration. Emergence of new viral diseases such as soybean dwarf virus, tobacco streak virus, and more recently soybean vein necrosis virus (SVNV) can further reduce yield. complicate the situation. *Soybean vein necrosis virus* is a species in the genus *Orthotospovirus* (Bunyavirales: Tospoviridae) and was first identified in Tennessee in 2008 (Tzanetakis et al., 2009). Since its discovery, SVNV has been documented in all the major soybean-growing in the United States and Ontario, Canada, including Arkansas, Illinois, Indiana, Iowa, Kentucky, Michigan, Minnesota, Ohio, Tennessee, Wisconsin (Bloomingdale et al., 2014). The symptoms begin with vein clearing followed by chlorosis or appearance of light-green to yellow blotchy patches near the main vein, followed by necrosis or dying of the leaf tissue at late stage of infection; hence, the name, SVNV.

Tospoviruses impact food and ornamental crops, encompassing hundreds of plant species, resulting in crop disease epidemics of economic significance worldwide (Pappu et al., 2009). Some members in this genus include, tomato spotted wilt virus (TSWV) and impatiens necrotic spot virus (INSV). As a member of the *Orthotospovirus* genus, SVNV is transmitted exclusively by thrips (Ullman et al., 1993). The thrips-*Orthotospovirus* relationship is unique since adult thrips are only able to transmit SVNV if acquisition occurs in the first instar and early second larval stages (Van De Wetering et al., 1996). This suggests that acquisition of the virus is an essential determinant of adult vector competency. Once virus is acquired by larval thrips, the virions enter the insect midgut (MG) epithelial cells, replicate and migrate to the muscle cells surrounding the midgut, and eventually reach and replicate in the salivary glands (Montero-Astúa et al., 2016). After infection of salivary glands, adults can release the virus into viable plant cells by the injection of viruliferous saliva during feeding. Infection of the principal salivary glands (PSG) is required for successful transmission (Kritzman et al., 2002), however, the route by which the virus reaches the salivary glands was not known. It was proposed that the virus may move from the MG to the salivary glands by direct contact between these tissues during early larval stages (Moritz et al., 2004); through the ligaments that connect each PSG to MG (Nagata et al., 1999; de Assis Filho et al., 2002); or the tubular salivary glands (TSG) that connect the MG directly to the PSG (Ullman et al., 1989). Recently, Montero-Astúa et al. (2016) showed that TSG and associated structures (e.g., Ligaments, efferent duct, filament-like structure) may serve as a channel for virus infection to progress from the MG to the PSG. The tospovirus membrane glycoproteins, G_N_ and G_C_ are involved in virus-binding and entry into thrips midgut epithelial cells (Bandla et al., 1998; Nagata et al., 2000; Whitfield et al., 2004). Presence of non-structural (NSs) protein in thrips is an indication of virus replication, whereas detection of nucleocapsid protein (NP) indicates presence or absence of virus in the insect tissues (Bandla et al., 1994). Tospoviruses are not transmitted transovarially (Wijkamp et al., 1996).

Currently there are 15 putative and 11 recognized tospovirus species with 15 thrips species in the family Thripidae that act as vectors (Rotenberg et al., 2015). There is considerable variation in thrips species ability to transmit tospoviruses (Jones, 2005). In a study, *F. occidentalis*, *F. schultzei*, *Thrips tabaci*, and *T. palmi* were evaluated for their competence to transmit four tospoviruses including, TSWV, tomato chlorotic spot virus (TCSV), groundnut ringspot virus (GRSV), and chrysanthemum stem necrosis virus (CSNV). *Frankliniella occidentalis* transmitted all four tospoviruses with different efficiencies. *Frankliniella schultzei* transmitted TCSV, GRSV, and CSNV, but *T. tabaci and T. plami* could not transmit any of the abovementioned viruses (Nagata et al., 2004). Srinivasan et al. (2012) showed that *T. tabaci* was a more efficient transmitter of IYSV compared to *F. fusca* (76.6 and 18.3% transmission rate, respectively). Transmission efficiencies can also vary within thrips populations or biotypes of the same species (Wijkamp et al., 1995; Chatzivassiliou et al., 2002; Nagata et al., 2004; Jacobson and Kennedy, 2013). For instance, the dark and pale forms of *F. schultzei* differ in their ability to transmit tospoviruses, with the dark form considered to be a more efficient vector (Wijkamp et al., 1995). These findings suggest that the transmission efficiency of a newly identified tospovirus in a specific area should be studied in the different populations of thrips species that live there.

Recently, soybean thrips, *Neohydatothrips variabilis* (Beach) was recognized as the primary vector of SVNV (Zhou and Tzanetakis, 2013; Keough et al., 2016). However, 2 years of thrips trapping in soybean fields in Indiana showed that there were as many as 8 different species including, *N*. *variabilis*, *Frankliniella tritici*, *F. fusca*, *Frankliniella* sp., *Thrips tabaci*, *Phlaeothrips* sp., *Anaphothrips* sp., and *Echinothrips* sp., Together, *N*. *variabilis*, *F. tritici*, and *F. fusca* account for nearly 75% of the thrips fauna found on soybean (Keough et al., 2018). Analysis of vector competence in the three thrips species revealed that tobacco thrips (*F. fusca*) and eastern flower thrips (*F. tritici*) are able to transmit SVNV albeit at a lower efficiency (36 and 6%, respectively) compared to the primary vector, soybean thrips (72%) (Keough et al., 2016). This was the first record of *F. tritici* as a vector of tospoviruses even though its vector competence to transmit other tospoviruses had been examined previously (Sakimura, 1953; de Assis Filho et al., 2005).

There has been progress in understanding various aspects of tospoviruses (Zhou et al., 2011; Khatabi et al., 2012; Zhou and Tzanetakis, 2013; Groves et al., 2016; Anderson et al., 2017), but many important details about virus acquisition, retention, and transmission by the vectors is are lacking. The aims of this study were to (1) elucidate the association virus acquisition [6, 12, 24, and 48 h acquisition access period (AAP)] and transmission efficiency [12, 24, and 48 h inoculation access period (IAP)] in the primary vector, *N. variabilis*, (2) understand mechanisms of vector competency by examining the effect of AAP (6, 12, and 24 h) on virus infection in the tissues of adult *N. variabilis* and (3) compare virus infection in the tissues all three vector species, *N. variabilis*, *F. tritici*, and *F. fusca*. Virus accumulation in adult insects was determined by absolute quantification method using Reverse Transcriptase-quantitative PCR (RT-qPCR) and transmission of virus to leaf tissues was determined by SVNV-specific double-antibody sandwich enzyme-linked immunosorbent assay (DAS-ELISA). Virus infection was determined by immunolabeling with antibody against SVNV-NP and imaged using confocal florescence microscopy.

## Materials and Methods

### Plant Source

Soybean variety Asgrow AG3334 (Monsanto, St. Louis, MO, United States) plants were grown in 15.24 cm pots at 25 ± 1°C with 60–70% relative humidity and a photoperiod of 16:8 (L:D) hours in an Environmental Control Room with IntellusUltra controller (Percival-Scientific, Perry, IA, United States). For plant maintenance, soybeans were fertilized weekly with Peters water-soluble fertilizer (14:14:14, N: P: K; Scotts-Sierra Horticultural Products, OH) at a rate of 3 g/l.

### Insect Source

Populations of *N. variabilis* originally collected from several different soybean fields located in Purdue Agricultural Centers (PAC) throughout Indiana were maintained in 45.72 × 45.72 × 76.20 cm thrips-proof insect cages (Bioquip, CA, United States) on soybean varAG3334. Despite several attempts, we could not transmit SVNV by mechanical-inoculation from which acquisition assays could be conducted. Other studies reported having similar difficulty (Khatabi et al., 2012; Hajimorad et al., 2015), hence we maintained infected and non-infected vector colonies in separate rooms. Insects were grouped into SVNV-infected and non-infected populations were distinguished by setting up thrips lines, where a single adult female was allowed to infest on a detached leaf at V1 or vegetative stage 1 (14 days after planting) in separate modified Petri-dishes with lids fitted with thrips-proof mesh (Bioquip, CA, United States) for ventilation. Infested leaves were tested for presence or absence of SVNV using RT-PCR after 2 weeks of incubation (Keough et al., 2016). All the offspring from thrips lines that tested positive for SVNV were pooled together to start an SVNV-infected colony, whereas the offspring from leaves that tested negative for SVNV were combined to start SVNV non-infected colony. SVNV-infected and non-infected colonies were maintained in 45.72 × 45.72 × 76.20 cm thrips-proof insect cages in separate rooms. Colonies were tested monthly using Diagnostic DAS-ELISA and RT-PCR for the presence of SVNV in plants and insects, respectively. Two other thrips species, *F. tritici* and *F. fusca*, were also collected from PAC locations and maintained on red kidney bean leaves (*Phaseolus vulgaris* L) in modified 50 ml Falcon tubes with a water source at the bottom and lids fitted with thrips-proof mesh for ventilation. Both *F. tritici* and *F. fusca* had higher fecundity on red bean compared to soybean, hence we used red bean for routine colony maintenance to obtain sufficiently large population needed for molecular analysis and confocal microscopy experiments. However, previous research in our lab showed that *F. tritici* and *F. fusca* can feed and reproduce on soybean albeit not as successfully as *N. variabilis* (Keough et al., 2016). All thrips populations were reared under the similar condition at temperature of 25 ± 1°C and a photoperiod of 16:8 (L:D) hours.

### Virus Source

SVNV was maintained in the greenhouse on soybean plants with continual transmission by *N. variabilis* at temperature of 25 ± 1°C and a photoperiod of 16:8 (L:D) hours. Disease-free, V3 stage of soybean plants were alternately added into SVNV-infected colony at 4–5 week intervals, allowing inoculation by viruliferous soybean thrips for 3–4 weeks in 45.72 × 45.72 × 76.20 cm thrips-proof cage at which time they were used as viral source in experiments. Plants were routinely checked for symptom development which typically took 3 weeks. Leaf tissues were harvested from symptomatic plants and infection was confirmed using SVNV-specific double-antibody sandwich enzyme-linked immunosorbent assay, DAS-ELISA kit (Agdia, Elkhart, IN, United States).

### SVNV Acquisition and Transmission by *N. variabilis* in Leaf-Disk Assay

The experimental design was a 4 × 3 factorial with four AAP durations (6, 12, 24, and 48 h) and three IAP durations (24, 48, and 72 h). Given the logistics of performing a complete factorial, we focused each experiment on a specific AAP duration and the cohort of thrips post-AAP were divided into three subsamples for the three inoculation treatments: 24, 48, and 72 h IAP, respectively. Briefly, a cohort of 50 first instars of *N. variabilis*, up to 24 h old, were placed on detached SVNV-infected soybean leaves for either 6, 12, 24, or 4 8h of AAP in 15 cm diameter Petri-dishes with lids fitted with thrips-proof mesh for ventilation. The petioles of infected detached soybean leaf were inserted through a hole made in the Parafilm cover on the top of a 1.7 ml microcentrifuge tubes, this was done to keep the soybean leaves fresh during the acquisition assay. All systemically infected leaves used for AAP were confirmed to be positive with SVNV. After varying duration of AAP, the cohort of larvae were transferred to non-infected/healthy soybean leaves sealed in Petri-dishes and reared to adulthood. The transmission efficiencies of thrips were assessed by inoculating a single 1.5 cm-diameter healthy soybean leaf disk with an individual thrips, 24 h post-adult eclosion. The soybean leaf disk was placed on a piece of filter paper partially inserted in 1000 μl micropipette tip, which was sealed off at one end and filled with water. Insects were allowed 24, 48, and 72 h of IAP, after which time insects were collected in 1.7 ml microcentrifuge tubes and flash frozen in liquid nitrogen (Supplementary Figure 1). Virus level in each insect was quantified using RT-qPCR estimated by log_10_ copies of SVNV NP RNA as calculated from an external standard dilution series of the plasmid containing the NP-gene (Keough et al., 2016). Leaf disks after each IAP were incubated on water in a microtiter plate for 4 days to allow for virus replication before being tested for SVNV with DAS-ELISA. Three negative controls (healthy soybean leaves), two blank buffer controls and three positive controls were included in each ELISA plate. Absorbance values were measured at 405 nm in a photometer 1 h after substrate addition. Since each experiment/ELISA plate is considered independent in order to compare absorbance values between plates, we expressed absorbance as a ratio by dividing the absorbance value of each sample/well by the average absorbance of the negative control wells of the corresponding plate. Thus, the ratio indicates how many times higher or lower was the sample value with respect to the corresponding plate negative controls. Samples with an absorbance value higher than three times the average of the negative controls were considered SVNV positive. This experiment was repeated four times for a total of 272 insects and corresponding leaf disks for 12 AAP × IAP (4 AAP × 3 IAP) treatment combinations. A cohort of larvae exposed to healthy leaf tissues served as the control group. Assays were conducted under laboratory conditions mentioned previously.

### Quantification of Virus in *N. variabilis* Adults Using Quantitative Real-Time-PCR

Total RNA was extracted from individual adult *N. variabilis* obtained from the acquisition and transmission assay described above using the Chelex 100 (Bio-Rad, Hercules, CA, United States) method (Boonham et al., 2002). A total of 11 μl of RNA was used for cDNA synthesis using the Verso cDNA^®^ Synthesis Kit (Thermo Fisher Scientific, Pittsburgh, PA, United States). To quantify SVNV levels from samples, a standard curve was generated using a plasmid containing SVNV-NP gene at a known concentration as per Keough et al. (2016). Briefly, a 239 bp SVNV-NP fragment was amplified using RT-PCR with the designed primer pair (forward: 5′-GGAAGCTTACCCCTTCTGGC-3′ and reverse: 5′-ACTCCTCTCATTTGGGGTGC-3′). The cycling conditions as follows: 2 min incubation at 94°C followed by 40 cycles of 30 s denaturation at 94°C, 10 s annealing at 55°C, and 1 min extension at 72°C and a final 10 min incubation at 72°C. The amplicon was cloned into pCR8^TM^ TA vector (2817 bp) using One Shot TOP10 chemically competent *Escherichia coli* (Thermo Fisher Scientific, Pittsburgh, PA, United States). Plasmid DNA was extracted using Qiagen Miniprep Kit following manufacturer’s protocol (Qiagen, Valencia, CA, United States) and sequenced. The nucleotide sequence was 100% identical to the target sequences deposited in GenBank (GU722322). The SVNV-NP sequence is deposited in NCBI GenBank under accession number MG190869. The concentration of the plasmid DNA containing the SVNV-NP gene was determined using a NanoDrop 1000 (Thermo Fisher Scientific, United States). The total plasmid size of 3056 bp was estimated by adding 239 bp of product size expected from the NP primers to 2817 bp of plasmid. The mass of the plasmid containing the NP gene was calculated using the following formula (Applied Biosystems, 2013), where m = mass and n = plasmid size (bp): m = (n) (1.096e^-21^g/bp). Ten-fold dilutions series, ranging from 7.54 × 10^8^ copies/μl to 7.54 × 10^1^ copies/μl, were prepared in RNase-free water for obtaining a standard curve. The qPCR Mastermix for SYBR Green^®^ (BioRad, Berkeley, CA, United States) was used according to manufacturer’s instructions. Each reaction contained 5.0 μl of SYBR Green Taq, 0.5 μl of F primer, 0.5 μl of R primer, 2.0 μl of deionized water, and 2.0 μl of cDNA template. The qPCR was run on a CFX Connect^®^ (BioRad, Berkeley, CA, United States) thermocycler, and the mean threshold cycles (C_q_) values were calculated by the CFX Manager^TM^ Software Version 3.1. The cycling conditions used were: 95°C for 2 min; followed by 40 cycles of 95°C for 10 s, 55°C for 30 s, and then the final melt curve, starting at 65°C increasing to 95°C, in increments of 0.5°C every 5 s. Each reaction plate contained a negative and positive control and standard curve along with insect samples, which were performed in duplicate. The number of SVNV RNA molecule copies in each PCR reaction was estimated by interpolating the normalized NP C_q_ value against the standard curve. A normalization coefficient (N_c_) was used for normalization of each NP C_q_ value, which was calculated from the internal control gene, Cytochrome C Oxidase Subunit I (COI), using the equation: Nc = 1 - (inC_q_ - MinC_q_)/MinC_q_., where inC_q_ is the C_q_ value of the internal control gene for a sample and MinC_q_ is the mean inC_q_ for all samples (Li et al., 2009). The primer pair for COI were 5′- GGATTTATTGTTTGAGCACACCAC-3′ and 5′-TCCTGTCAATCCTCCTAATGTGA-3′ (Harwood et al., 2007). The total copy number per thrips was calculated by multiplying the RNA dilution factor (9.1-fold) and then the cDNA template dilutions (40-fold). The amplification efficiency (E) was determined using the LinRegPCR software (Ruijter et al., 2009).

### Dissecting and Immunolabeling of Thrips Guts and Salivary Glands

Cohorts of 50 first instars of *N. variabilis* fed on SVNV-infected leaves for 6, 12, or 24 h AAP were reared on healthy soybean leaves until adulthood as described previously. Fifty *F. tritici* and *F. fusca* first instar larvae were allowed a 24 h AAP on infected soybean tissues as results from acquisition assays with *N. variablis* found no difference in virus acquisition rate post 12 and 24 h AAP (Table 1). Thrips were then reared to adulthood on healthy tissues. Thrips, 48 h post-adult eclosion, were placed in the middle of a Petri-dish encircled by 7% sucrose for 3 h of gut clearing. Thrips dissection and immunolabeling method were modified based on previously published research (Montero-Astúa et al., 2016). Ice-cold live thrips were decapitated under stereo dissecting microscope by cutting between the first and second thorax segments with #15 sterile scalpel blades (Microscopes America, Cumming, GA, United States) on a glass slide coated with poly-L-lysine (Thermo Fisher Scientific, Waltham, MA, United States). A drop of ice-cold PBS (10 μl) was added on the decapitated thrips and the abdomen was gently pressed using a minute pin to help obtaining the gut and salivary glands. The gut and salivary glands were removed from the remaining thrips body by cutting at the mesothorax. The organs were kept in PBS (100 μl) for 20 min at 4°C. After processing of the guts, PBS was removed carefully by pipetting and a 1 cm-diameter of circular incubation chamber was made around the guts using a glue gun. Slide was placed on ice for 1 min to avoid overheating and then kept at room temperature to allow the glue to dry. The incubation chamber was filled with 200 μl of 4% paraformaldehyde in 50 mM sodium phosphate (pH 7.2) and incubated for 1 h at room temperature in a humid box wrapped with aluminum foil to protect from the light and desiccation throughout the immunolabeling procedure. The slide was washed once with PBS and left overnight in humid box at 4°C with 200 μl of PBS-1% Triton X-100. All the subsequent procedures were conducted at room temperature in dark surroundings. The slides were washed three times with PBS and incubated in blocking buffer [PBS-0.1% Triton X-100 and 10% normal goat serum (NGS)] for 1 h. After blocking, the guts and salivary glands were washed once with PBS and incubated with 200 μl of alkaline phosphatase conjugated antibody against SVNV-NP at 1:200 dilution in ECl buffer (Agdia, Elkhart, IN, United States) for 2 h. The slides were washed three times with PBS for 5 min and incubated with sheep polyclonal anti-alkaline phosphatase antibody conjugated with FITC (Abcam, Cambridge, MA, United States) at 5 μg/ml in PBS-0.1% Triton X-100 and 1% NGS for 2 h. The slides were washed once with PBS and incubated for 1 h with 100 μl of phalloidin conjugated to iFluor 594 (Abcam, Cambridge, MA, United States) at 1:1000 dilution in PBS and 100 μl of DAPI (4′, 6-diamidino-2-phenylindole, dihydrochloride) at 125 μg/ml (Invitrogen, Grand Island, NY, United States). The slides were washed five times with PBS, and then rinsed three times with distilled water. The slides were air dried for 1 min after removal of the incubation chamber with a razor blade. A cover slide was carefully covered on the guts where 30 μl of PBS-50% glycerol was applied. Slides were sealed with clear nail polish and kept at 4°C in a microscope slide box.

**Table 1:** Detection of SVNV-NP protein in soybean leaf tissues in leaf-disk assays and corresponding SVNV-NP log copies in viruliferous adults.

AAP-IAP (h)	Number of insects tested	Number of positive leaf disks	Transmission rate (%)	Mean ±*SE* absorbance ratio of positive leaf disks	Mean ±*SE* SVNV-NP log copies in viruliferous insects
6–24	18	2	11.11	2.98 ± 0.005	2.99 ± 0.03
6–48	21	1	4.76	3.00 ± 0.00	3.21 ± 0.00
6–72	19	1	5.26	4.33 ± 0.00	6.00 ± 0.00
12–24	29	20	68.97	3.70 ± 0.46	3.52 ± 0.09
12–48	30	16	53.33	39.86 ± 9.97	3.65 ± 0.91
12–72	29	9	31.03	19.04 ± 5.49	3.21 ± 0.93
24–24	20	13	65.00	5.00 ± 1.51	3.53 ± 1.06
24–48	26	6	23.08	39.48 ± 17.65	4.32 ± 1.93
24–72	21	8	38.10	26.51 ± 9.37	3.95 ± 1.40
48–24	17	1	5.88	6.33 ± 0.00	3.12 ± 0.00
48–48	22	3	13.64	53.97 ± 31.16	3.67 ± 2.12
48–72	20	4	20.00	60.79 ± 30.39	2.36 ± 1.18

*First instar larvae (up to 24 h old) were exposed to SVNV-infected soybean leaves for varying duration of Acquisition Access Period (AAP). Thrips were then reared to adulthood on uninfected soybean leaves and given varying duration of Inoculation Access Periods (IAP) on uninfected soybean leaf disks. Individual leaf disks were assayed for SVNV infection by DAS-ELISA to determine the percentage of SVNV-positive leaves or transmitting adults. Viral load per insect was estimated by log_10_ copies of SVNV-NP RNA per insect as calculated from an external standard dilution series of the plasmid containing the SVNV NP-gene.*

### Confocal Fluorescent Microscopy

SVNV accumulation in guts and salivary glands were examined by Olympus Fluoview^®^ FV10i Confocal Laser Scanning Microscopy (Olympus, Tokyo, Japan) using a B-2A filter and a 60X oil-immersion objective with variable scan zoom. Images were exported using Olympus FV10-ASW Version 3.1 and further processed with ImageJ Version 1.45. The pinhole aperture was fixed at ×2.0 μm for all three channels. The laser intensity and sensitivity of light absorption for specific wavelengths were set as follow: a 405 nm laser diode at 30% of sensitivity and 30% of laser intensity for detecting blue DAPI fluorescence; a 473 nm laser diode at 45% of sensitivity and 60% of laser intensity for detecting green FITC fluorescence; and a 559 nm laser diode at 45% of sensitivity and 55% of laser intensity for detecting red Alexa Fluor 594. Z-stacks were taken for every image for all three channels with an automatic calculated optimum of 1.00 μm per slide. The depth of each insect tissue for z-stack imaging was manually adjusted and set to the highest and lowest points of the observed feature, which were determined when one of the extremes was barely perceptible. Gain settings for green channel were fixed as mentioned above to compare the intensity of SVNV signal between images. Gain for the other two channels was adjusted accordingly to obtain the best detail of nuclei and actin in each image.

### Quantitative Analysis of Fluorescence Signal in PSG

To quantify SVNV infection in the PSG, Image J software (Ansari et al., 2012) was used to analyze the FITC fluorescence signal. Images attained from green FITC fluorescence channel were used for measuring area, mean intensity and integral density (IntDen). Each image was converted to an 8-bit gray image after removal of the default scale. The area of interest in each image was confined with freehand selection tool for measurement. The regions with no fluorescence were also selected for obtaining readings for background. SVNV infection in PSG was evaluated by computing the mean intensity of fluorescence signal (MIS). MIS was calculated using the following formula: MIS = IntDen - (Area of selected tissue × Mean fluorescence of background readings).

### Statistical Analyses

Virus transmission (ELISA absorbance ratios) was treated as a binomial response (positive or negative), and differences were estimated by logistic regression analysis by using Proc GENMOD with logit link function in SAS 9.4 (SAS Institute, 2013) as per Srinivasan et al. (2012). Spearman’s rank correlation coefficients (rho) were calculated to determine correlation between virus transmission (binomial response) in the ELISA assays and SVNV log_10_ copies in corresponding insects. Analyses of percentage of SVNV-positive tissues by immunolabeling was performed using Chi-square test. A one-way ANOVA was performed to determine differences in the MIS in *N. variabilis* PSG after 6, 12, and 24 h AAP, and between MIS in *F. fusca and F. tritici* PSG. All statistical analyses were performed using Minitab^®^ Version 16 (Minitab Stat College, PA, United States).

## Results

### Acquisition and Transmission Efficiency of *N. variabilis*

**Figure 1:**
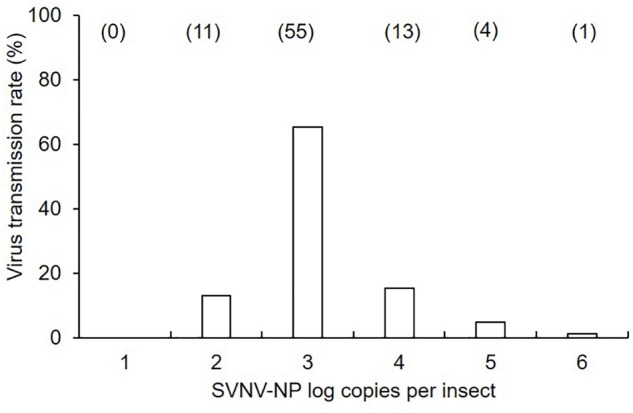
Mean of soybean vein necrosis virus log copies in *N. variabilis* adults that exhibited successful virus transmission in leaf-disk assays. The number of SVNV-NP RNA in individual adults was categorized into each order from < 10^1^ to 10^6^. Numbers in parentheses above each bar represent the number of *N. variabilis* in each virus titer category. Transmission rate was calculated by dividing the number of individuals per category by total number of positive insects (*n* = 84).

There was a significant effect of AAP (χ^2^ = 24.85; *df* = 3; *P* < 0.0001), but not IAP (χ^2^ = 0.34; *df* = 2; *P* = 0.845) on virus transmission (binomial response -positive or negative) in leaf disks using ELISA (Table 1). The transmission rate was significantly different between 6 and 12 h AAP (*P* = 0.001), 6 and 24 h AAP (*P* = 0.001), 12 and 48 h AAP (*P* = 0.008), and 24 and 48 h AAP (*P* = 0.007). No difference in transmission rate was observed between 6 and 48 h AAP (*P* = 0.267) and 12 and 24 h AAP (*P* = 0.860). Interestingly, transmission rate was low at 6 and 48 h AAP despite the relatively high accumulation of virus in insect tissues (Table 1). No correlation was detected between virus transmission rate (binomial response -positive or negative) and SVNV-NP RNA levels in corresponding *N. variabilis* adults (rho = -0.21, *P* = 0.73). SVNV transmission occurred at varying levels of virus RNA accumulation in insect tissues ranging from > 10^2^ to ≤ 10^6^ (Figure 1). The highest transmission rate was observed at 10^3^ copies of viral RNA per insect and transmission reduced in adults harboring higher accumulation of the virus (>10^4^ to ≤ 10^6^) (Figure 1).

### Immunodetection of SVNV in *N. variabilis* Guts and Salivary Glands

**Figure 2:**
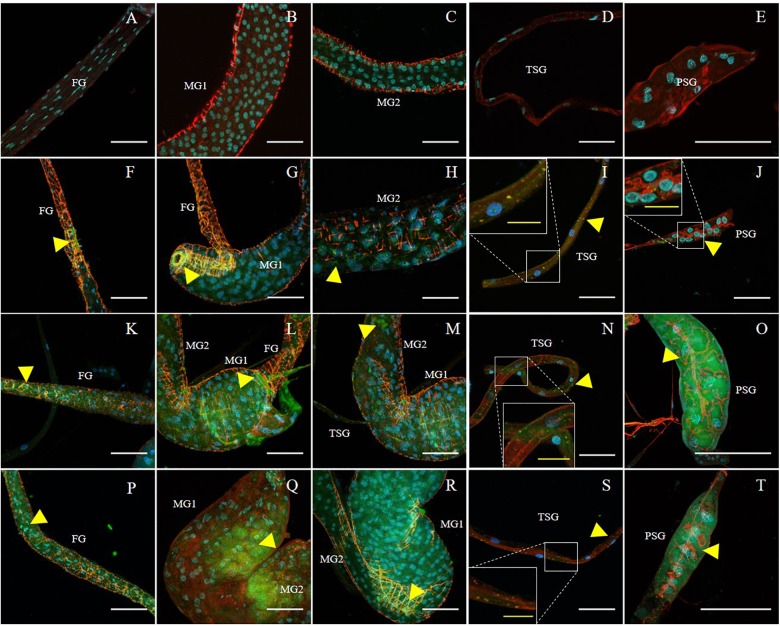
Immunodetection of SVNV in guts and salivary glands of *N. variabilis* adults. Localization of viral particles in the foregut (FG), midgut region 1 (MG1), midgut region 2 (MG2), principal salivary gland (PSG), and tubular salivary glands (TSG) was observed by confocal fluorescence microscopy with FITC conjugated secondary antibody against the primary antibody targeting SVNV-NP. The control for the labeling was SVNV-free or uninfected adult *N. variabilis* **(A–E)**. SVNV infection in adults post 6 h AAP **(F–J)**, post 12 h AAP **(K–O)**, and post 24 h AAP **(P–T)**. Green, blue and red fluorescences indicate presence of SVNV-NP, nuclei (DAPI) and actin (phalloidin conjugated to iFluor 594), respectively. Yellow arrows indicate intense fluorescent signal. White scale bar (50 μm) and yellow scale bar (20 μm).

The presence of SVNV virions in alimentary canal and salivary glands of adult *N. variabilis* after 6, 12, and 24 h AAP was detected using indirect immunofluorescence technique with primary antibody against SVNV-NP and FITC fluorophore-conjugated secondary antibody targeting the primary antibody and imaged using confocal fluorescent microscopy. Thrips fed on SVNV-free soybean leaves were used as negative control (Figure 2A–E). A total of 70 thrips were evaluated and 57 (81%) were considered SVNV-positive due to the detection of fluorescent signals in the various tissues. SVNV infection of each tissue from adult *N. variabilis* after varying AAP is summarized in Table 2. In adult *N. variabilis*, strong signals were detected in the foregut (FG), with high frequency of FG infection (>50%) at 12 and 24 h AAP. Foregut infection was only observed when MG1 was infected, indeed there was a significant association between FG and MG1 infection at 6-h AAP (χ^2^ = 11.045, *df* = 1, *P* = 0.001), but not at 12 and 24 h AAP (χ^2^ = 1.296, *df* = 1, *P* = 0.255 and χ^2^ = 1.279, *df* = 1, *P* = 0.258, respectively). Among three sections of midgut (MG1, MG2 and MG3), the highest percentage of infection was observed in MG1 followed by MG2 and MG3 (Table 2). Infection of the PSG was observed when the TSG were infected, however, the correlation was not statistically significant at either AAPs.

**Table 2:** Immunodetection of SVNV in adult *N. variabilis* exposed to varying Acquisition access period (AAP) on SVNV-infected leaf tissues.

AAP (h)	Percentage of infection					
	FG	MG1	MG2	MG3	TSG	PSG
6	37 (10/27)	81 (22/27)	42 (11/26)	27 (3/11)	32 (6/19)	15 (2/13)
12	67 (14/21)	82 (18/22)	79 (15/19)	44 (4/9)	71 (12/17)	67 (6/9)
24	65 (11/17)	81 (17/21)	75 (12/16)	56 (5/9)	53 (8/15)	54 (7/13)

*SVNV-positive tissues were determined by observation of FITC green fluorescence signal, which indicates the existence of SVNV-NP. Percent infection was determined for each tissue examined and the numbers in parenthesis are the number of positive tissues observed over the total number of tissues examined. The counts for some tissues from certain samples were not recorded because of the broken or missing of the fragile tissues or structures caused by dissecting and immunolabeling processes. FG, Foregut; MG1, midgut region 1; MG2, midgut region 2; MG3, midgut region 3; PSG, principal salivary gland; TSG, tubular salivary gland.*

Frequency of SVNV infection in individual tissues including FG, MG1, MG2 and MG3, TSG, and PSG varied with AAP duration (Figure 2A–D,F–T and Table 2). At 6 h AAP, significantly lower virus infection was found in FG (χ^2^ = 5.847, *df* = 1, *P* = 0.016), MG2 (χ^2^ = 6.041, *df* = 1, *P* = 0.014), TSG (χ^2^ = 7.795, *df* = 1, *P* = 0.005) and PSG (χ^2^ = 7.052, *df* = 1, *P* = 0.008) compared to 12 h AAP (Table 2). Of the infection between 6 and 24 h AAP, significantly lower infection was found in MG2 (χ^2^ = 4.102, *df* = 1, *P* = 0.043) and PSG (χ^2^ = 4.248, *df* = 1, *P* = 0.039). The percentage of SVNV-infected tissues were similar between 12 and 24 h AAP (Table 2). Overall, the length of AAP did not affect virus infection in MG1, however, significantly lower infection was observed in FG, MG2, and PSG post 6 h AAP.

**Figure 3:**
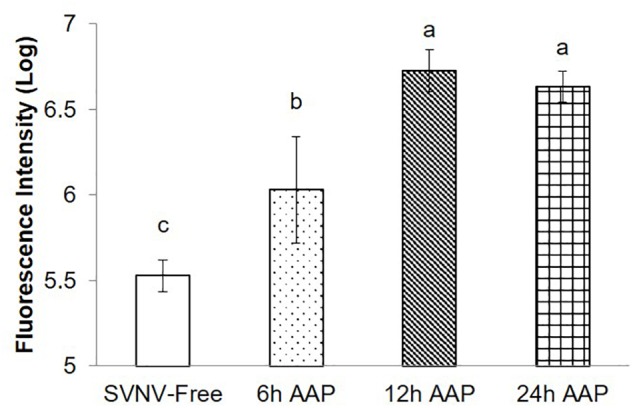
Mean intensity of fluorescence signal (MIS) in PSG of *N. variabilis* after varying time of AAP. Means that do not share a letter are significantly different.

Given the significance of PSG infection in virus transmission, we quantified PSG infection indirectly by the MIS. We found significantly lower amount and extent of infection at 6 h AAP compared to 12 and 24 h AAP, but higher than SVNV-free control (Figure 3, *P* < 0.0001). It was not uncommon that SVNV infection was not detectable in PSG at 6 h AAP (Figure 2J). There was no significant difference of MIS in PSG between 12 and 24 h AAP (Figure 3). Overall, results of the confocal experiments mirror the transmission assay, in that lowest virus transmission was observed at 6 h AAP, which also had lowest frequency of SVNV infection in the various tissues. In addition, the frequency of infected tissues was largely similar between 12 and 24 h AAP and no significant difference in transmission rate was observed in the leaf-disk assay using ELISA (Tables 1, 2).

### Immunodetection of SVNV in *F. tritici* and *F. fusca* Guts and Salivary Glands

**Figure 4:**
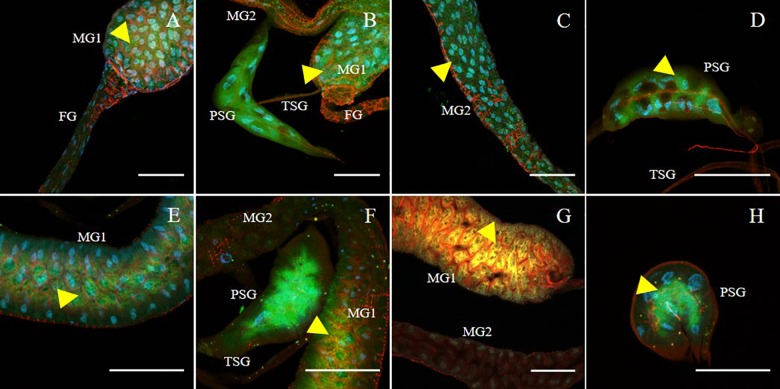
Immunodetection of SVNV in guts and salivary glands of *F. fusca and F. tritici* adults. Localization of viral particles in the foregut (FG), midgut region 1 (MG1), midgut region 2 (MG2), principal salivary gland (PSG) and tubular salivary glands (TSG) was observed by confocal fluorescence microscopy with FITC conjugated secondary antibody against the primary antibody targeting SVNV-NP. SVNV infection in adult *F. fusca* **(A–D)** and *F. tritici* **(E–H)** post 24 h AAP. The control for the labeling was SVNV-free or uninfected adult of each species (Data not shown). Green, blue and red fluorescences indicate presence of SVNV-NP, nuclei (DAPI) and actin (phalloidin conjugated to iFluor 594), respectively. Yellow arrows indicate intense fluorescent signal. Scale bar indicates 50 μm.

**Figure 5:**
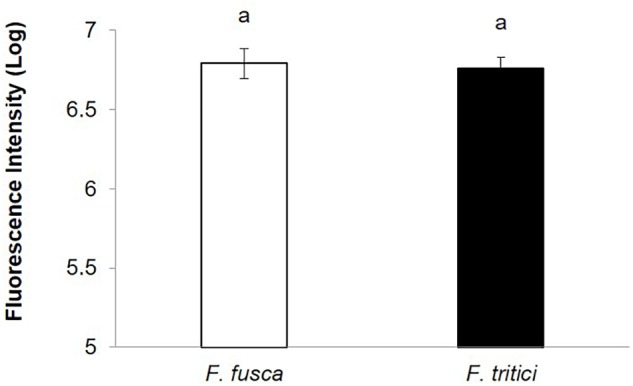
Mean intensity of fluorescence signal (MIS) in PSG of secondary vectors, *F. fusca*, and *F. tritici* after 24 h AAP. Means that do not share a letter are significantly different.

SVNV accumulation in adult *F. fusca* and *F. tritici* after 24 h AAP was investigated using the same immunolabeling procedure described for *N. variabilis*. A total of 22 *F. fusca* and 23 *F. tritici* were tested, among which 15 (68%) and 6 (26%) were considered SVNV-positive, respectively. The percentage of infection of each tissue for *F. fusca* and *F. tritici* is summarized in Table 3. Signals of virus infection was detected in all the examined tissues in *F. fusca* and *F. tritici* except for MG3 in *F. tritici* (Table 3 and Figure 4). The FG showed high frequency of infection in *F. fusca* but low infection in *F. tritici* (Table 3 and Figure 4). Consistent with the pattern observed in *N. variabilis* (Table 2), the MG1 showed the highest infection rate among the three regions of midgut in both *F. fusca* and *F. tritici* (Table 3). Unlike *N. variabilis*, where no significant association existed for SVNV infection between FG and MG1, we found a significant association between FG and MG1 in *F. fusca* and *F. tritici* (χ^2^ = 4.792, *df* = 1, *P* = 0.029 and χ^2^ = 3.972, *df* = 1, *P* = 0.046, respectively). The extremely low infection rate of PSG in *F. tritici* (9%) very likely contributed to their inefficiency for virus transmission. The MIS in PSG of *F. tritici* was lower than *F. fusca* after 24 h AAP but the difference was not significant (*P* = 0.822) (Figure 5).

**Table 3:** Immunodetection of SVNV in adult *Frankliniella fusca* and *F. tritici* exposed to 24 h Acquisition access period (AAP) on infected leaf tissues.

Species	Percentage of infection					
	FG	MG1	MG2	MG3	TSG	PSG
*F. fusca*	32 (6/19)	68 (15/22)	56 (10/18)	43 (6/14)	21 (3/14)	36 (5/14)
*F. tritici*	9 (2/22)	26 (6/23)	15 (3/20)	0 (0/17)	27 (4/15)	9 (2/22)

*SVNV-positive tissues were determined by observation of FITC green fluorescence signal, which indicates the existence of SVNV-NP. Percent infection was determined for each tissue examined and the numbers in parenthesis are the number of positive tissues observed over the total number of tissues examined. The counts for some tissues from certain samples were not recorded because of the broken or missing of the fragile tissues or structures caused by dissecting and immunolabeling processes. FG, Foregut; MG1, midgut region 1; MG2, midgut region 2; MG3, midgut region 3; PSG, principal salivary gland; TSG, tubular salivary gland.*

## Discussion

The viral load or number of virus RNA in transmitting adult thrips is considered a key determinant of virus transmission. For example, Nagata et al. (2002) found that adults of *F. occidentalis* that transmitted TSWV efficiently harbored higher titers of the virus compared to *T. tabaci* that failed to transmit the virus. Another study reported a positive correlation between transmission efficiency of thrips and TSWV titer in host plants (Okazaki et al., 2011). Rotenberg et al. (2009) found that not only was TSWV titer an important factor for vector competence, but virus titer was positively associated with frequency of transmission events. In contrast to the TSWV-thrips system, we did not detect a significant correlation between virus transmission rate (binomial response -positive or negative) measured by ELISA absorbance ratios and SVNV-NP RNA levels in corresponding *N. variabilis* adults. Indeed, some thrips having high virus titer (10^4^–10^5^) had similar transmission abilities like those harboring less virus titer (10^2^). Similar result was reported for southern rice black-streaked dwarf virus accumulation in *Sogatella furcifera* adults (Matsukura et al., 2015). One possibility to explain this reduced transmission could be the failure of virus infection of the salivary glands or limited virus accumulation in these glands despite the vectors harboring high virus titer in their gut and other tissues. Also, extensive accumulation of viruses sometimes has negative impact on vector insects, such as lower feeding activity and reduced fitness (Robb, 1989; DeAngelis et al., 1993; Matsukura et al., 2015). Previously, we showed that fecundity of *N. variabilis* decreased with increasing viral titer (Keough et al., 2016). It is possible that excessive accumulation of SVNV might result in a lower virus transmission rate because of lower fecundity resulting from lower feeding activity of *N. variabilis*. Research is underway in our laboratory to determine the effect of viral load on probing activity of male and female thrips using the Electrical Penetration Graph (EPG) technique. Moreover, quantification of virus titer in salivary glands could be more informative because infection of the salivary glands is a requirement for virus transmission to occur. Nevertheless, knowledge of viral titer in the vector is a useful indicator to manage virus epidemics in the field.

Analysis of SVNV-NP protein revealed the presence of the virus in the foregut (FG), midgut (MG), tubular salivary gland (TSG) and principal salivary gland (PSG) of adult *N. variabilis.* We found that the MG had highest virus infection compared to other tissues. In adults that had high percentage of PSG infection (12 and 24 h AAP), the first region of the midgut (MG1) had slightly greater percentage of infection than that observed in the second and third regions of the midgut (MG2 and MG3, respectively). It has been reported that midgut regions have distinct morphological, histological, and genetic properties (Buchon et al., 2013; Marianes and Spradling, 2013; O’Brien Lucy, 2013), which may result in different regions being more or less competent to virus infection. We also observed a high frequency of FG infection at 12 and 24 h AAP (67 and 65%, respectively). In contrast, Montero-Astúa et al. (2016) reported ≤30% infection of FG in *F. occidentalis* adults post 18 h AAP on TSWV-infected tissues as first instars. It is believed that the infection first begins in the first larval instar by infecting MG1, followed by infection of midgut muscle cells, the TSG and the efferent duct that leads to the PSG. In adults, infection could spread from MG1 to the FG, MG2, and MG3 from previously infected tissues such as ligaments that connect at the junction of the FG and MG1, by cell-to-cell movement through midgut epithelial cells and/or midgut muscle cells (Nagata et al., 1999; Kritzman et al., 2002). Hence, the primary site of virus infection is shifted from MG1 and TSG in larvae to PSG in adults (Montero-Astúa et al., 2016). Our results reveal a high frequency of SVNV infection in FG and MG1 and MG2 of adult soybean thrips that showed consistent PSG infection. Since we did not analyze virus infection in larval stages, we cannot make conclusions about changes in tissue tropism from larvae to adults. Nevertheless, the importance of secondary amplification of the virus in other organs outside the MG before reaching the PSG needs further investigation. Analysis of *N. variabilis* adults where the PSG and TSG were intact showed co-occurrence of virus infection in the two tissues albeit not statistically significant. This confirms previous reports that TSG serve as conduit for virus movement from MG1 to the PSG in adult thrips (Ullman et al., 1992; Nagata et al., 1999; Montero-Astúa et al., 2016). Overall, there appears to be similarities and differences between the TSWV-*F. occidentalis* system and SVNV- *N. variabilis* system. Viral proteins were detected in similar organs in *F. occidentalis* and *N. variabilis*, and adults with consistent PSG infection are likely to have corresponding TSG infected. *N. variabilis* adults that had high PSG infection also had high percentage of FG and MG2 infection, whereas FG and MG2 infection was low in *F. occidentalis* adults. This suggest greater lateral cell migration of virions in *N. variabilis* compared to *F. occidentalis* adults. Lastly, both MG1 and PSG were heavily infected in *N. variabilis* adults, whereas PSG appear to be primary site of virus infection in adult *F. occidentalis*.

Based on our previous results from acquisition and transmission assays, we wanted to investigate the effect of AAP on SVNV accumulation in alimentary canal and salivary glands of *N. variabilis*. Our analysis revealed that most *N. variabilis* adults became infected after AAP of 6, 12, or 24 h with the infection found in all organs being studied. Interestingly, the length of AAP did not affect virus infection in MG1, however, significantly lower infection was observed in FG, MG2, and PSG post 6 h AAP. In a previous study, there was no effect of AAP on MG infection in adult *F. occidentalis*, however, the percentage of infected vesicular muscles cells was lower in thrips fed on infected plants for 3 h than those which had fed for 16 h AAP (Nagata et al., 1999). The percentage of PSG infection was significantly lower for *N. variabilis* adults that acquired the virus as larvae after 6 h AAP than 12 and 24 h AAP, where infection rates of PSG were nearly 2.5 and 1.7 times higher, respectively. This suggests that there are significant differences in development of infection in larval tissues when given shorter time of virus acquisition. Since transmission efficiency is highly dependent on the degree of infection of PSG, the partial and/or weak infection of PSG at 6 h AAP could result in much lower transmission rate than the actual infection rate of PSG (Nagata et al., 1999). Overall, results from the transmission assays and immunolabeling experiments suggest that shorter AAP results in reduced virus infection in the various tissues especially in the PSG which could be due to the restriction of virus to the midgut and/or failure to disseminate from the midgut to PSG, which subsequently results in reduced transmission efficiency.

Two secondary vector species*, F. fusca* and *F. tritici* that fed on SVNV-infected tissue for 24 h as first instar larvae were able to acquire and transmit the virus as indicated by detection of viral proteins (N) in individual thrips using confocal fluorescence microcopy. However, the frequency of infected tissues was lower in *F. fusca* and *F. tritici* compared to the primary vector, *N. variabilis*. These results are in concordance with our previously published work where we found presence of non-structural NSs proteins in adult *F. fusca* and *F. tritici* after feeding on virus-infected material, which confirmed virus replication in these insects (Keough et al., 2016). The pattern of virus accumulation in *N. variabilis*, *F. fusca*, and *F. tritici* was similar except for lack of virus infection in MG3 of *F. tritici*. SVNV was also detected in TSG and PSG of both secondary vectors, which supports the theory that TSG serve as potential route for tospoviruses to infest PSG in thrips vectors. Our data showed that SVNV is able to infect PSG of *F. tritici* after 24 h AAP on virus-infected material. A previous study did not detect TSWV-NSs protein in PSG, ligaments, TSG, and hindgut of *F. tritici* (de Assis Filho et al., 2005). Unlike TSWV, it appears that SVNV has evolved the ability to be acquired and transmitted by a previous non-vector of tospovirus, *F. tritici*. Overall, results of our study identified similarities and differences in virus transmission mechanisms compared to the well-studied TSWV-*F. occidentali*s system. There appears to be distinct associations between thrips species and their ability to transmit tospoviruses (Jones, 2005), hence it is important to assess vector competence in each thrips species. Future research should be aimed at tissue-specific analysis of thrips-encoded proteins that mediate virus replication which would greatly improve our knowledge of molecular interaction between the virus and vector and identify novel targets for development of transgenic technologies to limit the ability of insect vectors to transmit plant pathogens.

## Author Contributions

PN conceived and designed the experiments, analyzed the data, and wrote the manuscript. JH performed the experiments, analyzed the data, and wrote a draft of the manuscript. VN analyzed the data and revised the manuscript. I-CY provided training for use of the confocal microscope. All authors read and approved the final manuscript.

## Conflict of Interest Statement

The authors declare that the research was conducted in the absence of any commercial or financial relationships that could be construed as a potential conflict of interest.

## References

[B1] Anderson N. R. Irizarry M. D. Bloomingdale C. A. Smith D. L. Bradley C. A. Delaney D. P. (2017). Effect of *Soybean vein necrosis* on yield and seed quality of soybean. *Can. J. Plant Pathol.* 39 334–341. 10.1080/07060661.2017.1354333

[B2] Ansari N. Müller S. Stelzer E. Pampaloni F. (2012). Quantitative 3D cell-based assay performed with cellular spheroids and fluorescence microscopy. *Methods Cell Biol.* 113 295–309. 10.1016/B978-0-12-407239-8.00013-6 23317907

[B3] Applied Biosystems (2013). *Creating Standard Curves with Genomic DNA or Plasmid DNA Templates for use in Quantitative PCR.* Foster, CA: Applied Biosystems 1–8

[B4] Bandla M. D. Westcot D. M. Chenault K. Ullman D. E. German T. L. Sherwood J. (1994). Use of monoclonal antibody to the nonstructural protein encoded by the small RNA of Tomato spotted wilt tospovirus to identify viruliferous thrips. *Phytopathology* 84 1427–1431. 10.1094/Phyto-84-1427

[B5] Bandla M. D. Campbell L. R. Ullman D. E. Sherwood J. L. (1998). Interaction of *Tomato spotted* wilt tospovirus (TSWV) glycoproteins with a thrips midgut protein, a potential cellular receptor for TSWV. *Phytopathology* 88 98–104. 10.1094/PHYTO.1998.88.2.98 18944977

[B6] Bloomingdale C. Bradley C. Chilvers M. Giesler L. Groves R. Mueller D. (2014). *Soybean Vein Necrosis Virus. Soybean Disease Management.* Available at: http://www.soybeanresearchinfo.com/pdf_docs/SVNV_CPN1003_2015.pdf

[B7] Boonham N. Smith P. Walsh K. Tame J. Morris J. Spence N. (2002). The detection of *Tomato spotted* wilt virus (TSWV) in individual thrips using real time fluorescent RT-PCR (TaqMan). *J. Virol. Methods* 101 37–48. 10.1016/S0166-0934(01)00418-9 11849682

[B8] Buchon N. Osman D. David F. P. Fabrice P. A. Yu Fang H. Boquete J.-P. (2013). Morphological and molecular characterization of adult midgut compartmentalization in Drosophila. *Cell Rep.* 3 1725–1738. 10.1016/j.celrep.2013.04.001 23643535

[B9] Chatzivassiliou E. K. Peters D. Katis N. I. (2002). The efficiency by which *Thrips tabaci* populations transmit *Tomato spotted* wilt virus depends on their host preference and reproductive strategy. *Phytopathology* 92 603–609. 10.1094/PHYTO.2002.92.6.603 18944256

[B10] de Assis Filho F. M. Naidu R. A. Deom C. M. Sherwood J. L. (2002). Dynamics of *Tomato spotted* wilt virus replication in the alimentary canal of two thrips species. *Phytopathology* 92 729–733. 10.1094/PHYTO.2002.92.7.729 18943268

[B11] de Assis Filho F. M. Stavisky J. Reitz S. R. Deom C. M. Sherwood J. L. (2005). Midgut infection by *Tomato spotted* wilt virus and vector incompetence of *Frankliniella tritici*. *J. Appl. Entomol.* 129 548–550. 10.1111/j.1439-0418.2005.01006.x

[B12] DeAngelis J. Sether D. Rossignol P. (1993). Survival, development, and reproduction in western flower thrips (Thysanoptera: Thripidae) exposed to impatiens necrotic spot virus. *Environ. Entomol.* 22 1308–1312. 10.1093/ee/22.6.1308

[B13] Groves C. German T. Dasgupta R. Mueller D. Smith D. L. (2016). Seed transmission of *Soybean vein necrosis* virus: the first tospovirus implicated in seed transmission. *PLoS One* 11:e0147342. 10.1371/journal.pone.0147342 26784931 PMC4718560

[B14] Hajimorad M. Halter M. Wang Y. Staton M. Hershman D. (2015). Evaluation of seed transmissibility of *Soybean vein necrosis*-associated virus in two soybean cultivars grown under field conditions. *J. Plant Pathol. Microbiol.* 6:278.

[B15] Harwood J. D. Desneux N. Yoo H. J. S. Rowley D. L. Greenstone M. H. Obrycki J. J. (2007). Tracking the role of alternative prey in soybean aphid predation by *Orius insidiosus*: a molecular approach. *Mol. Ecol.* 16 4390–4400. 10.1111/j.1365-294X.2007.03482.x 17784913

[B16] Jacobson A. L. Kennedy G. G. (2013). Specific insect-virus interactions are responsible for variation in competency of different *Thrips tabaci* isolines to transmit different *Tomato spotted* wilt virus isolates. *PLoS One* 8:e54567. 10.1371/journal.pone.0054567 23358707 PMC3554729

[B17] Jones D. R. (2005). Plant viruses transmitted by thrips. *Eur. J. Plant Pathol.* 113 119–157. 10.1007/s10658-005-2334-1

[B18] Keough S. Danielson J. Marshall J. Lagos D. Voegtlin D. Srinivasan R. (2018). Factors affecting population dynamics of thrips vectors of Soybean vein necrosis virus. *Environ. Entomol.* 47 734–740. 10.1093/ee/nvy021 29506040

[B19] Keough S. Han J. Shuman T. Wise K. Nachappa P. (2016). Effects of *Soybean vein necrosis* virus on life history and host preference of its vector, *Neohydatothrips variabilis*, and evaluation of vector status of *Frankliniella tritici* and *Frankliniella fusca*. *J. Econ. Entomol.* 109 1979–1987. 10.1093/jee/tow145 27417640

[B20] Khatabi B. Wen R. H. Hershman D. Kennedy B. Newman M. Hajimorad M. (2012). Generation of polyclonal antibodies and serological analyses of nucleocapsid protein of *Soybean vein necrosis*-associated virus: a distinct soybean infecting tospovirus serotype. *Eur. J. Plant Pathol.* 133 783–790. 10.1007/s10658-012-9969-5

[B21] Kritzman A. Gera A. Raccah B. Van Lent J. Peters D. (2002). The route of *Tomato spotted* wilt virus inside the thrips body in relation to transmission efficiency. *Arch. Virol.* 147 2143–2156. 10.1007/s00705-002-0871-x 12417949

[B22] Li W. Abad J. A. French-Monar R. D. Rascoe J. Wen A. Gudmestad N. C. (2009). Multiplex real-time PCR for detection, identification and quantification of ’Candidatus Liberibacter solanacearum’ in potato plants with zebra chip. *J. Microbiol. Methods* 78 59–65. 10.1016/j.mimet.2009.04.009 19409423

[B23] Marianes A. Spradling A. C. (2013). Physiological and stem cell compartmentalization within the Drosophila midgut. *eLife* 2:e00886. 10.7554/eLife.00886 23991285 PMC3755342

[B24] Matsukura K. Towata T. Yoshida K. Sakai J. Okuda M. Onuki M. (2015). Quantitative analysis of southern rice black-streaked dwarf virus in *Sogatella furcifera* and virus threshold for transmission. *Phytopathology* 105 550–554. 10.1094/PHYTO-05-14-0142-R 25870927

[B25] Montero-Astúa M. Ullman D. E. Whitfield A. E. (2016). Salivary gland morphology, tissue tropism and the progression of tospovirus infection in *Frankliniella occidentalis*. *Virology* 493 39–51. 10.1016/j.virol.2016.03.003 26999025

[B26] Moritz G. Kumm S. Mound L. (2004). Tospovirus transmission depends on thrips ontogeny. *Virus Res.* 100 143–149. 10.1016/j.virusres.2003.12.022 15036845

[B27] Nagata T. Almeida A. C. L. Resende R. O. DeÁvila A. C. (2004). The competence of four thrips species to transmit and replicate four tospoviruses. *Plant Pathol.* 53 136–140. 10.1111/j.0032-0862.2004.00984.x

[B28] Nagata T. Inoue-Nagata A. K. Prins M. Goldbach R. Peters D. (2000). Impeded thrips transmission of defective *Tomato spotted wilt virus* isolates. *Phytopathology* 90 454–459. 10.1094/PHYTO.2000.90.5.454 18944549

[B29] Nagata T. Inoue-Nagata A. K. Smid H. M. Goldbach R. Peters D. (1999). Tissue tropism related to vector competence of *Frankliniella occidentalis* for *Tomato spotted* wilt tospovirus. *J. Gen. Virol.* 80 507–515. 10.1099/0022-1317-80-2-507 10073714

[B30] Nagata T. Inoue-Nagata A. K. van Lent J. Goldbach R. Peters D. (2002). Factors determining vector competence and specificity for transmission of *Tomato spotted* wilt virus. *J. Gen. Virol.* 83 663–671. 10.1099/0022-1317-83-3-663 11842261

[B31] O’Brien Lucy E. (2013). Regional specificity in the drosophila midgut: setting boundaries with stem cells. *Cell Stem Cell* 13 375–376. 10.1016/j.stem.2013.09.008 24094316 PMC3897550

[B32] Okazaki S. Okuda M. Komi K. Yamasaki S. Okuda S. Sakurai T. (2011). The effect of virus titre on acquisition efficiency of *Tomato spotted* wilt virus by *Frankliniella occidentalis* and the effect of temperature on detectable period of the virus in dead bodies. *Aust. Plant Pathol.* 40 120–125. 10.1007/s13313-010-0020-z

[B33] Pappu H. Jones R. Jain R. (2009). Global status of tospovirus epidemics in diverse cropping systems: successes achieved and challenges ahead. *Virus Res.* 141 219–236. 10.1016/j.virusres.2009.01.009 19189852

[B34] Robb K. L. (1989). *Analysis of Frankliniella Occidentalis (Pergande) as a Pest of Floricultural Crops in California Greenhouses.* Doctoral Dissertation, University of California, California.

[B35] Rotenberg D. Jacobson A. L. Schneweis D. J. Whitfield A. E. (2015). Thrips transmission of tospoviruses. *Curr. Opin. Virol.* 15 80–89. 10.1016/j.coviro.2015.08.003 26340723

[B36] Rotenberg D. Krishna Kumar N. K. Ullman D. E. Montero-Astúa M. Willis D. K. German T. L. (2009). Variation in *Tomato spotted wilt virus* titer in *Frankliniella occidentalis* and its association with frequency of transmission. *Phytopathology* 99 404–410. 10.1094/phyto-99-4-0404 19271982

[B37] Ruijter J. M. Ramakers C. Hoogaars W. M. H. Karlen Y. Bakker O. van den Hoff M. J. B. (2009). Amplification efficiency: linking baseline and bias in the analysis of quantitative PCR data. *Nucleic Acids Res.* 37:e45. 10.1093/nar/gkp045 19237396 PMC2665230

[B38] Sakimura K. (1953). *Frankliniella tritici*, a non-vector of the spotted wilt virus. *J. Econ. Entomol.* 46 915–916. 10.1093/jee/46.5.915

[B39] SAS Institute Inc (2013). *SAS^^®^^ 9.4.* Cary, NC: SAS Institute Inc.

[B40] Soystats (2017). *2017 Soy Highlights.* Available at: http://www.soystats.com/2012/ [accessed October 4 2017].

[B41] Srinivasan R. Sundaraj S. Pappu H. R. Diffie S. Riley D. G. Gitaitis R. D. (2012). Transmission of iris yellow spot virus by *Frankliniella fusca* and *Thrips tabaci* (Thysanoptera: Thripidae). *J. Econ. Entomol.* 105 40–47. 10.1603/EC11094 22420253

[B42] Tzanetakis I. Wen R. Newman M. Hajimorad M. (2009). *Soybean vein necrosis* virus: a new threat to soybean production in southeastern united states. *Phytopathology* 99:S131.

[B43] Ullman D. German T. Sherwood J. Westcot D. Cantone F. (1993). Tospovirus replication in insect vector cells: immunocytochemical evidence that the nonstructural protein encoded by the S RNA of *Tomato spotted* wilt tospovirus is present in thrips vector cells. *Phytopathology* 83 456–463. 10.1094/Phyto-83-456

[B44] Ullman D. E. Cho J. J. Mau R. F. L. Westcot D. M. Custer D. M. (1992). A midgut barrier to *Tomato spotted* wilt virus acquisition by adult western flower thrips. *Phytopathology* 82 1333–1342. 10.1094/Phyto-82-1333

[B45] Ullman D. E. Westcot D. M. Hunter W. B. Mau R. F. L. (1989). Internal anatomy and morphology of *Frankliniella occidentalis* (Pergande) (Thysanoptera: Thripidae) with special reference to interactions between thrips and *Tomato spotted* wilt virus. *Int. J. Insect Morphol. Embryol.* 18 289–310. 10.1016/0020-7322(89)90011-1

[B46] Van De Wetering F. Goldbach R. Peters D. (1996). *Tomato spotted* wilt tospovirus ingestion by first instar larvae of *Frankliniella occidentalis* is a prerequisite for transmission. *Phytopathology* 86 900–905. 10.1094/Phyto-86-900

[B47] Whitfield A. E. Ullman D. E. German T. L. (2004). Expression and characterization of a soluble form of *Tomato spotted wilt virus* glycoprotein G_N_. *J. Virol.* 78 13197–13206. 10.1128/JVI.78.23.13197-13206.2004 15542672 PMC524983

[B48] Wijkamp I. Almarza N. Goldbach R. Peters D. (1995). Distinct levels of specificity in thrips transmission of tospoviruses. *Phytopathology* 85 1069–1074. 10.1094/Phyto-85-1069

[B49] Wijkamp I. Goldbach R. Peters D. (1996). Propagation of *Tomato spotted* wilt virus in *Frankliniella occidentalis* does neither result in pathological effects nor in transovarial passage of the virus. *Entomol. Exp. Appl.* 81 285–292. 10.1046/j.1570-7458.1996.00098.x

[B50] Zhou J. Kantartzi S. K. Wen R. H. Newman M. Hajimorad M. R. Rupe J. C. (2011). Molecular characterization of a new tospovirus infecting soybean. *Virus Genes* 43 289–295. 10.1007/s11262-011-0621-9 21604150

[B51] Zhou J. Tzanetakis I. (2013). Epidemiology of *Soybean vein necrosis* associated virus. *Phytopathology* 103 966–971. 10.1094/PHYTO-12-12-0322-R 23550970

