# Supplementary figures and images for: Vector Competence of Thrips Species to Transmit Soybean Vein Necrosis Virus
Source: Front Microbiol. 2019 Mar 19;10:431. doi: 10.3389/fmicb.2019.00431 (PMC6433834; doi:10.3389/fmicb.2019.00431)

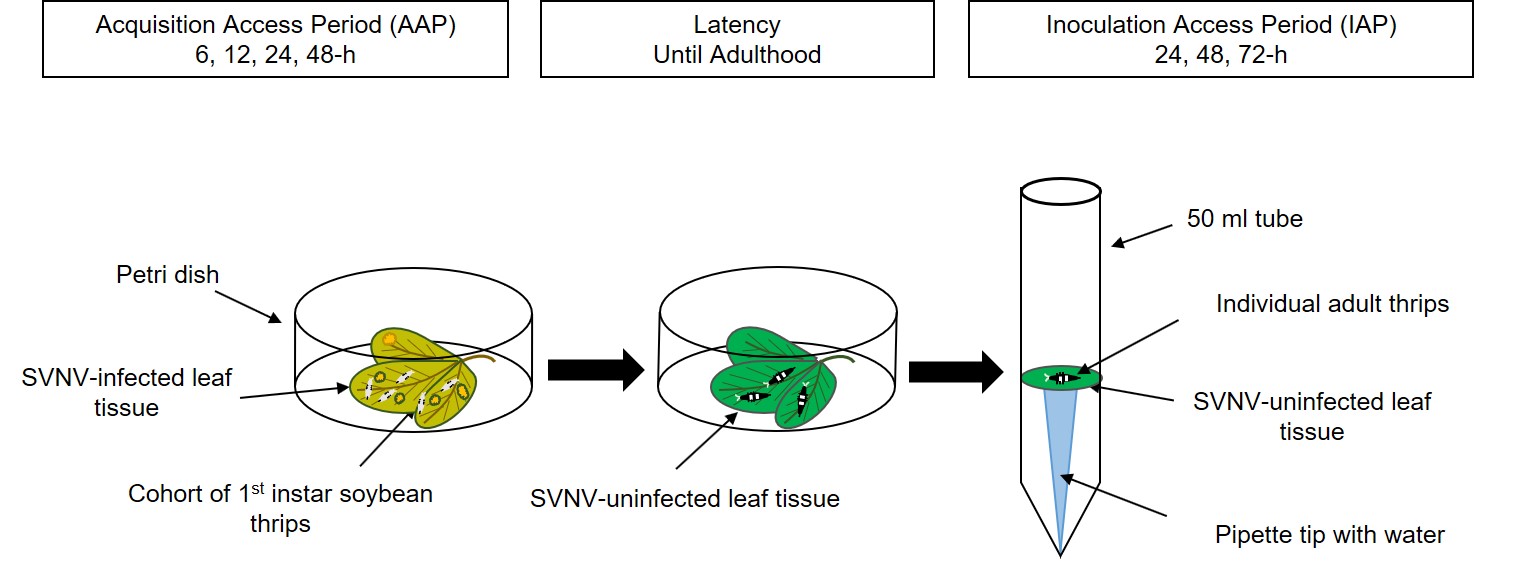

Supplement: Supplementary file 1 [file Image_1.JPEG]
